# Insula neuroanatomical networks predict interoceptive awareness

**DOI:** 10.1016/j.heliyon.2023.e18307

**Published:** 2023-07-18

**Authors:** Alan S.R. Fermin, Takafumi Sasaoka, Toru Maekawa, Hui-Ling Chan, Maro G. Machizawa, Go Okada, Yasumasa Okamoto, Shigeto Yamawaki

**Affiliations:** a Center for Brain, Mind and Kansei Sciences Research, Hiroshima University, 734-8553, Hiroshima city, Hiroshima, Japan; b Department of Psychiatry and Neurosciences, Hiroshima University, 734-8553, Hiroshima city, Hiroshima, Japan

**Keywords:** Insula, Interoception, Interoceptive awareness, Active inference, Brain interoception network

## Abstract

Interoceptive awareness (IA), the subjective and conscious perception of visceral and physiological signals from the body, has been associated with functions of cortical and subcortical neural systems involved in emotion control, mood and anxiety disorders. We recently hypothesized that IA and its contributions to mental health are realized by a brain interoception network (BIN) linking brain regions that receive ascending interoceptive information from the brainstem, such as the amygdala, insula and anterior cingulate cortex (ACC). However, little evidence exists to support this hypothesis. In order to test this hypothesis, we used a publicly available dataset that contained both anatomical neuroimaging data and an objective measure of IA assessed with a heartbeat detection task. Whole-brain Voxel-Based Morphometry (VBM) was used to investigate the association of IA with gray matter volume (GMV) and the structural covariance network (SCN) of the amygdala, insula and ACC. The relationship between IA and mental health was investigated with questionnaires that assessed depressive symptoms and anxiety. We found a positive correlation between IA and state anxiety, but not with depressive symptoms. In the VBM analysis, only the GMV of the left anterior insula showed a positive association with IA. A similar association was observed between the parcellated GMV of the left dorsal agranular insula, located in the anterior insula, and IA. The SCN linking the right dorsal agranular insula with the left dorsal agranular insula and left hyper-granular insula were positively correlated with IA. No association between GMV or SCN and depressive symptoms or anxiety were observed. These findings revealed a previously unknown association between IA, insula volume and intra-insula SCNs. These results may support development of non-invasive neuroimaging interventions, e.g., neurofeedback, seeking to improve IA and to prevent development of mental health problems, such anxiety disorders.

## 1 Introduction

Interoception, the neural sensing of internal states of the body, including visceral, e.g., heart rate, blood pressure, and physiological signals, e.g., energy metabolism, thermoregulation, immune signals, is processed in the brain by specialized regions located in the cortex, such as the insula and ACC, as well as in subcortical and brainstem nuclei [[1], [2], [3]]. Ascending interoceptive signals, ranging from imperceptible molecules to heartbeats, oftentimes reach interoceptive awareness (IA), the subjective and conscious experience of visceral and physiological processes [[4], [5], [6]]. For instance, visceral signals reach awareness by their sensory stimulation of the body, such as pain, speed of heartbeats, inflation and deflation of the lungs and peristaltic movements of the gastrointestinal tract, whereas some chemical interoceptive signals reach awareness over longer time-scales via their influences on emotional processes, such as the emotion of hunger elicited by low glucose levels and hunger hormones [2]. IA has multiple facets that can be measured during objective tasks that assess an individual's accuracy in detecting body signals, e.g., heartbeat detection, or via subjective questionnaires that assess an individual's sensibility to self-report body signals [[7], [8], [9], [10]]. Despite recent progress in understanding the neural basis of interoception and IA, as well as their influences on cognitive processes and mental health [1,8,[11], [12], [13], [14], [15], [16]], whether IA is linked with distinct neuroanatomical structures or with neuroanatomical networks has yet to be fully understood.

The functions and structures of multiple subcortical and cortical brain regions have been associated with IA [1,6,12,14,[17], [18], [19]]. The amygdala, ACC and insula are among the brain regions implicated in IA and their mutual connections give rise to a large-scale brain interoceptive network (BIN) [16]. These regions receive interoceptive input from multiple brainstem nuclei with primary involvement in autonomic control of visceral and physiological functions [1,6,20]. In contrast to the amygdala and ACC, the insula is characterized by a hierarchical organization of its modular cytoarchitectures, namely, granular, dysgranular and agranular modules [[20], [21], [22], [23]]. The posterior granular insula is the major input region of ascending visceral information via the thalamus and propagates it to the middle dysgranular insula, and from there to the anterior agranular insula [20]. The insula also makes parallel connections with the prefrontal cortex (PFC) and the basal ganglia, structures implicated in higher-order cognitive processes, such as reasoning and introspection, adaptive behavior, inhibition and valuation of body, and environmental information [24,25]. This pattern of local cytoarchitectonic organization and neuroanatomical connections suggests that the insula implements specialized functions in IA, although their exact natures remain obscure.

We recently suggested that a better strategy to reveal insula functions is to understand the functions of the brain regions with which it forms anatomical connections [6]. A similar network approach, for instance, has been used to reveal the cognitive, motivational, and computational functions of the basal ganglia and cerebellum, previously thought to play restricted roles in motor control [[26], [27], [28], [29]]. Given its unique modular, hierarchical, and parallel neuroanatomical network organization, we have advanced the Insula Modular Active Control (IMAC) model [6] based on the active inference framework and predictive coding [[30], [31], [32]] to suggest that insula neuroanatomical networks, especially its networks with brain regions that also receive ascending interoceptive information (such as the amygdala and ACC), support active interoceptive inference and IA. According to the IMAC model, the three distinct modules of the insula and their connections with the PFC employ generative interoceptive models to predict and achieve desired visceral and physiological states, in order to reduce interoceptive prediction errors ascending from the body or arriving from other brain regions, e.g., the amygdala and ACC. In the IMAC model, the granular insula uses visceral-based representations to generate habitual-like interoceptive predictions, whereas the granular and agranular insula use higher-order interoceptive representations to introspect on the possible causes of the ascending interoceptive prediction errors and to generate behaviors and appropriate interoceptive predictions to fix those errors. However, evidence supporting the hypothesis that intra-insula, insula-amygdala or insula-ACC neuroanatomical networks contribute to IA is still lacking.

In the present study, in order to test the hypothesis on the contributions of the neuroanatomical connections of the insula to IA, we took advantage of a publicly available dataset [15] that contained anatomical neuroimaging data acquired with magnetic resonance imaging (MRI) and individual accuracy scores of IA assessed with a heartbeat detection task (HDT). In the HDT, higher accuracy scores are taken as an index of higher IA about cardiac signals, whereas low interoceptive accuracy scores indicate lower cardiac IA. Whole-brain multiple regression voxel-based morphometry (VBM) analyses were performed to investigate the neuroanatomical representation of IA. VBM was also used to estimate structural covariance networks (SCN), which measure individual differences in the strength of anatomical gray matter volume covariation between two brain regions. Pearson correlations were then used to investigate the association between SCN and IA. Since interoceptive signals also exert significant influences on human behavior, higher-order cognitive processes, and mental health, including the development of psychiatric disorders [4,6,14,15,[33], [34], [35], [36], [37], [38], [39], [40]], we also investigated whether the neuroanatomical representation of IA was also associated with self-reported depressive symptoms and anxiety.

## 2 Materials and methods

### 2.1 Participants

Behavioral and neuroimaging data used in the present study were downloaded from the open online database OpenNEURO (www.openneuro.org). Below we describe the portion of the data used in the present study and the neuroimaging and statistical analyses performed. Further details about the original dataset can be found at the following link (www.openneuro.org/datasets/ds003763/versions/1.0.5) and from the original paper [15].

The online dataset contained data and demographic information from 62 participants, predominantly of aged females (n = 44), aged males (n = 6), young females (n = 6), and young males (n = 6). Following inspection of the structural T1-weighted MRI data, only 40 participants (37 aged females; 2 aged males; 1 young female) had good quality structural data. Bad quality data included suboptimal defacing which led to cutting large portions of the brain. Damaged data, possibly due to errors during the neuroimaging acquisition process or while loading data into the online database, was also found. Since the original published paper which used this dataset, focused on aged females, we also decided to analyze the data of our final sample of 37 aged females (43–62 years), mean age, 52.6 years old.

### 2.2 Experimental paradigms

Participants performed 16 alternating 20-s blocks of a heartbeat detection task (HDT, 8 blocks) and a sound detection task (8 blocks) inside the MRI scanner. Participants received pre-training outside and inside the scanner prior to the main scanning session. In the sound task, participants had to press a button to indicate detection of a beep stimulus delivered through headphones, whereas in the HDT, participants pressed a button to indicate their own heartbeats. Control steps were taken to deliver beeps with a frequency and variance similar to heart rate. An interoception accuracy index was used to estimate cardiac IA in the HDT [41]. Interoceptive accuracy scores used in the present study are available in the original dataset found in the online database (www.openneuro.org/datasets/ds003763/versions/1.0.5).

### 2.3 MRI data acquisition

As described in the original paper, “MRI was performed with a Siemens MAGNETOM Verio 3T scanner (Erlangen, Germany) located at the Research Center of Neurology. A three-dimensional structural image consisted of a sagittal T1-weighted 3D-MPRAGE sequence (TR 1900 msec, TE 2.47 msec, voxel size 1 × 1 × 1 mm, FOV 250 mm)” [15].

### 2.4 MRI data analysis

The Computational Anatomy Toolbox (CAT12, http://dbm.neuro.uni-jena.de/cat/) and Statistical Parametric Mapping software (SPM12, http://www.fil.ion.ucl.ac.uk/spm) were used to preprocess structural T1-weighted images. A CAT12 default pipeline was used to pre-process structural images that were bias-corrected, tissue-classified (gray matter, white matter and cerebral spinal fluid), registered using linear (12-parameter affine) and non-linear transformations (warping), and modulated normalized. Modulated normalized images were smoothed in SPM12 with a full-width at half-maximum smoothing kernel of 12 × 12 × 12 mm.

The CAT12 toolbox was also used to estimate individual values of gray matter volume (GMV), white matter volume (WMV), cerebral spinal fluid (CSF), and total intracranial volume (TIV). CAT12 automatically performed region-based morphometry analysis to parcellate and estimate GMV of brain regions in native space using anatomical landmarks, based on the Brainnetome Atlas (BNA) [42]. Further details of this anatomical parcellation can be found in the CAT12 software. In the analyses described below, we were especially interested in the estimated GMV of the insula (six sub-regions per hemisphere, a total of 12 regions), ACC (two sub-regions per hemisphere, a total of four regions) and amygdala (two sub-regions per hemisphere, a total of four regions).

A whole-brain multiple regression voxel-based morphometry (VBM) analysis was conducted to investigate brain regions associated with IA, controlling for effects of age and TIV and an absolute threshold of 0.2. Given our hypothesis on the possible link between IA with the volumes of the amygdala, insula and ACC, results were considered significant below a statistical threshold of P < 0.001, uncorrected for the whole brain, followed by small volume correction (SVC) with family-wise error (FWE) and a statistical threshold of P_FWE_ < 0.05.

**Fig. 1:**
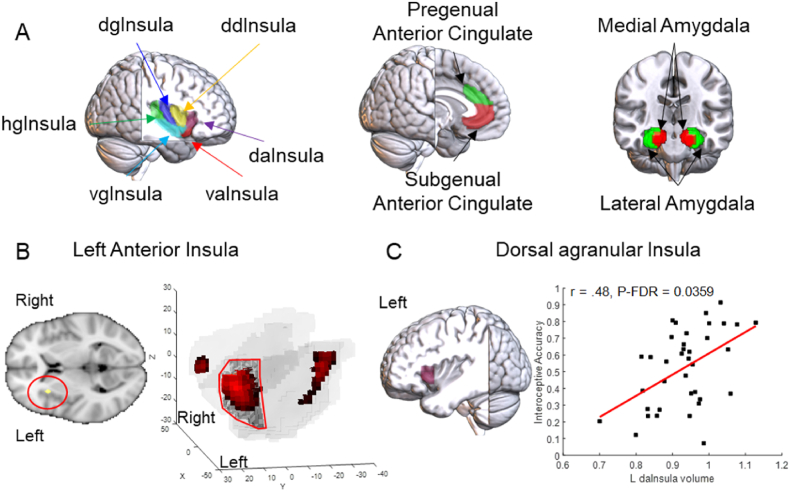
Insula volume representation of interoceptive awareness. (A) Brain regions of interest with their volumes parcellated based on the Brainnetome Atlas (BNA). hgInsula (hyper-granular insula), dgInsula (dorsal granular insula), vgInsula (ventral granular insula), ddInsula (dorsal dysgranular insula), daInsula (dorsal agranular insula), vaInsula (ventral agranular insula). (B) Right: the left anterior insula was the only region of interest with volume positively associated with IA. Left: For visualization purposes, voxels with T-values >2.5 within the insula were extracted and are displayed overlayed onto a bilateral insula mask image. The shaded gray area surrounding the left anterior insula cluster represents the area 167 (dorsal agranular area) of the Brainnetome Atlas (C) The parcellated volume of the dorsal agranular area is positively correlated with IA, consistent with the result in (B) panel.

In order to reliably demonstrate the relationship between the GMV of the brain regions of interest, we performed a series of Pearson correlations using parcellated volumes of the insula, ACC and amygdala (Fig. 1A). The association between the volumes of these regions and IA were considered significant after false discovery rate (FDR) correction with a statistical threshold of P_FDR_ < 0.05 for all sub-regions of each region of interest. These analyses controlled for the effects of age and TIV. An additional generalized linear multiple regression analysis was conducted to identify a region of interest with the highest predictability of IA. This regression model included the assessed IA (interoceptive accuracy scores) as the response and the GMV of all 20 sub-regions as predictors, while also controlling for the effects of age and TIV.

Structural Covariance Networks (SCN) were used to estimate individual differences in the strength of anatomical connectivity based on volume covariation between two brain regions [[43], [44], [45], [46], [47]]. SCNs were estimated with whole-brain multiple regression VBM analyses performed using the SPM12 software. Since only the volume of the left anterior insula (BNA area 167: left dorsal agranular insula) showed a significant association with IA (see Results section for statistical details), we decided to investigate only the SCNs of each insula sub-region (12 regions in total, six regions per hemisphere), as defined by the Brainnetome Atlas [42]. Thus, we conducted 12 distinct multiple regression VBM models, in which each model used the parcellated GMV of one (seed) of the 12 sub-regions of the insula as a covariate in order to identify voxels in the brain covarying with the volume of the seed region. All 12 VBM regression models included age and TIV as confounding factors. We focused these analyses on the identification of intra-insula, insula-amygdala and insula-ACC covariance networks.

In each of the 12 regression models, a SCN of an insula seed with the other insula, the amygdala, or ACC sub-regions, was considered significant if it met a whole-brain statistical threshold of P < 0.001, uncorrected, followed by a SVC and statistical threshold of P_FWE_ < 0.05 with the use of a mask image of the target region. Next, we used mask images of each identified region (amygdala, ACC, and insula), forming an SCN with an insula seed in whole-brain VBM analyses, to extract the non-adjusted average eigenvariate of all voxels in the mask. This eigenvariate signal was then used as an index of individual differences of the anatomical covariance connectivity strength with the seed region. The extracted anatomical connection strength values were used in Pearson correlations to investigate their relationship with IA, state anxiety, trait anxiety, and depression, while controlling for effects of age and TIV.

Finally, Pearson correlations were conducted to investigate the relationship between IA and self-reported depressive symptoms, state anxiety, and trait anxiety, controlling for the effects of age. See the original publication for details on assessment of depressive symptoms and anxiety [15]. Scores of depressive symptoms, state and trait anxiety from two participants were missing, leaving data of 35 participants for these correlations.

## 3 Results

### 3.1 Relationship between interoceptive awareness, depressive symptoms, and anxiety

IA was significantly positively correlated with state anxiety (r = .39, P = 0.0224). No association was observed between IA and trait anxiety (r = 0.021, P = 0.9060) or depressive symptoms (r = 0.035, P = 0.8433). These correlations controlled for effects of age.

Since previous studies have reported an association between age and reduced IA in heartbeat-counting tasks [48,49], we performed further analyses to identify whether the same relationship might be observed in our sample. In our aged female sample, the age of participants (mean = 52.59 years old, min = 43 years old, max = 62 years old, range = 19 years) showed no correlation with IA (r = −0.17, P = 0.2986), state anxiety (r = 0.07, P = 0.6881), trait anxiety (r = 0.15, P = 0.3932) or depressive symptoms (r = 0.18, P = 0.2986). Furthermore, linear regression models (See Table S1) revealed no significant effect of age on the relationship between IA and state anxiety (Age: beta = −0.0094, standard error = 0.0069, t-stat = −1.3658, P = 0.1815), IA and trait anxiety (Age: beta = −0.0084, standard error = 0.0076, t-stat = −1.1104, P = 0.2751), or IA and depressive symptoms (Age: beta = −0.0085, standard error = 0.0076, t-stat = −1.1232, P = 0.2697).

### 3.2 Neuroanatomical representation of interoceptive awareness

Whole-brain multiple regression VBM analysis revealed a significant positive association between IA and the volume of the left anterior insula (P_SVC-FWE_ = 0.011, cluster size = 149 voxels) (Fig. 1B, Table S2). Neither the volume of the amygdala nor ACC showed an association with IA.

Analysis of parcellated volumes of the sub-regions of the amygdala, insula, and ACC revealed a positive correlation between IA and the volume of the left anterior insula (dorsal agranular insula: r = 0.49, P_unc_ = 0.0029, P_FDR_ = 0.0359, corrected for the 12 insula sub-regions) (Fig. 1C). No other sub-regions of the insula, amygdala, and anterior cingulate showed a significant relationship with IA (Table S3). Consistent with the VBM and parcellated GMV analyses, the multiple regression model also showed that among all 20 sub-regions investigated, the GMV of the left dorsal agranular insula was the best predictor of IA (beta = 3.0144, standard error = 1.0674, T-value = 2.8240, P = 0.0135) (Table S4).

### 3.3 Insula structural covariance networks and interoceptive awareness

**Fig. 2:**
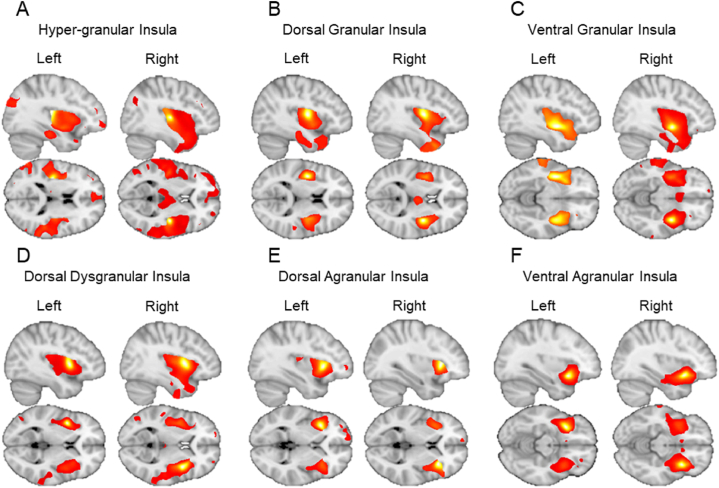
Whole-brain statistical maps of SCNs of each insula sub-region by hemisphere. (A) SCN of the hyper-granular insula (BNA areas 163 and 164). (B) SCN of the dorsal granular insula (BNA areas 171 and 172). (C) SCN of the ventral granular insula (BNA areas 169 and 170). (D) SCN of the dorsal dysgranular insula (BNA areas 173 and 174). (E) SCN of the dorsal agranular insula (BNA areas 165 and 166). (F) SCN of the ventral agranular insula (BNA areas 167 and 168). All images are thresholded at P < 0.001, uncorrected.

Based on previous studies [46,47], we identified insula SCNs if the gray matter volume of an insula seed region showed significant positive association with the gray matter volume of another brain region in a whole-brain VBM analysis. We focused our analyses on identification of intra-insula, insula-amygdala, and insula-ACC SCNs. In all conducted 12 multiple regression models, no significant negative associations were observed between insula seeds and other sub-regions the insula, amygdala or ACC. Fig. 2 shows the statistical parametric brain maps of the SCN of the hyper-granular insula (Fig. 2A), dorsal granular insula (Fig. 2B), ventral granular insula (Fig. 2C), dorsal dysgranular insula (Fig. 2D), dorsal agranular insula (Fig. 2E) and ventral agranular insula (Fig. 2F) (Table S5 for anatomical coordinates of main clusters).

We identified 155 statistically significant insula SCNs, which represent 67.98% of all possible SCNs (estimated at 228) (See Table S6 for the connection matrix; Table S7 for estimated percentages and Table S8 for small volume correction statistics). The intra-insula SCNs represented 81.29% of the identified SCNs, whereas the insula-ACC SCNs and the insula-amygdala SCNs represented 7.74% and 10.97%, respectively. When the percentages of SCNs are calculated based on insula modular granularity (granular, dysgranular and agranular), we found that the granular insula (hyper-granular, dorsal granular and ventral granular) formed 85 significant SCNs, representing 54.84% of all identified insula SCNs, followed by the dysgranular insula with 27 SCNs (17.42%) and the agranular insula (dorsal and ventral) with 43 (24.74%) significant SCNs.

**Fig. 3:**
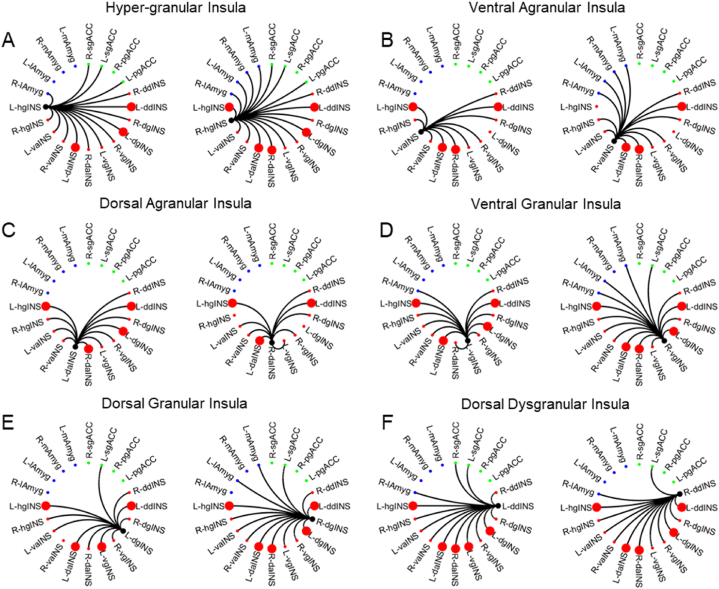
Intra-insula, insula-amygdala, and insula-anterior cingulate SCNs. (A) An SCN of the left and right hyper-granular insula. (B) An SCN of the left and right ventral-agranular insula. (C) An SCN of the left and right dorsal agranular insula. (D) An SCN of the left and right ventral granular insula. (E) An SCN of the left and right dorsal granular insula. (F) An SCN of the left and right dorsal dysgranular insula. Black lines indicate the identified SCN (see Fig. 2) that survived P < 0.001 uncorrected, followed by SVC with P_FWE_ < 0.05. Large red circles indicate the SCNs positively correlated with interoceptive awareness (Two-tailed Pearson correlations and P < 0.05, uncorrected, controlling for age and TIV). L: left. R: Right. hgINS: hyper-granular insula. vaINS: ventral agranular insula. daINS: dorsal agranular insula. vgINS: ventral granular insula. dgINS: dorsal granular insula. ddINS: dorsal dysgranular insula. (For interpretation of the references to colour in this figure legend, the reader is referred to the Web version of this article.)

**Fig. 4:**
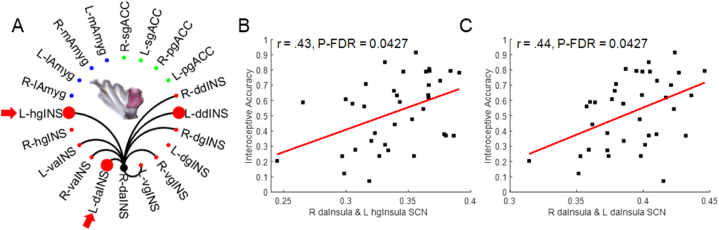
Right dorsal agranular insula SCN linked with interoceptive awareness. (A) Right dorsal agranular insula SCNs (black lines) and SCNs linked with interoceptive awareness (large red circles). Among all SCN linked with interoceptive awareness displayed in Fig. 3, only the SCNs of the right dorsal agranular insula with the left hyper-granular insula (B) and left dorsal agranular insula (C) survived a False Discovery Rate (FDR) test for multiple comparisons (also indicated by red arrows in panel A). L: left. R: Right. hgINS: hyper-granular insula. vaINS: ventral agranular insula. daINS: dorsal agranular insula. vgINS: ventral granular insula. dgINS: dorsal granular insula. ddINS: dorsal dysgranular insula. (For interpretation of the references to colour in this figure legend, the reader is referred to the Web version of this article.)

Among all identified insula SCNs, 50 (21.93%) were significantly positively correlated with IA (Fig. 3, Table S9) and all of these were intra-insula connections: eight hyper-granular insula SCNs (Fig. 3A); seven ventral agranular insula SCNs (Fig. 3B); seven dorsal agranular insula SCNs (Fig. 3C); nine ventral granular insula SCNs (Fig. 3D); nine dorsal granular insula SCNs (Figs. 3E) and 10 dorsal dysgranular insula SCNs (Fig. 3F). No insula-amygdala or insula-ACC connections were associated with IA. Granular insula SCNs represented 52% (26 SCNs) of the connections positively correlated with IA, followed by the dorsal dysgranular insula with 20% (10 SCNs) and the agranular insula with 28% (14 SCNs). However, after performing a FDR test for multiple comparisons, only two intra-insula SCNs remained significantly positively associated with IA (Fig. 4A). The SCN of the right dorsal agranular insula with the left hyper-granular insula (r = 0.43, P_unc_ = 0.0095, P_FDR_ = 0.0427, Fig. 4B) and with the left dorsal agranular insula (r = 0.43, P_unc_ = 0.0084,P_FDR_ = 0.0427, Fig. 4C) remained significantly positively associated with IA.

## 4 Discussion

In the present study we tested a hypothesis that the BIN, especially intra-insula networks and its networks with other interoceptive brain regions, contribute to IA. Our initial whole-brain VBM analysis revealed that among the amygdala, insula, and ACC, only the GMV of the left anterior insula was positively correlated with IA. This finding was further supported by analyses of the parcellated GMV and a regression model that revealed that higher IA was also positively associated with the larger volume of the left dorsal agranular area, a region located in the anterior insula and overlapping with the insula voxel cluster identified in the VBM analysis (Fig. 1B). Despite the positive association between anxiety and IA in the present study, a finding consistent with previous reports [7,50,51], no association between anxiety levels and GMV or SCNs of the insula were observed.

The present findings linking larger GMV of the left anterior insula with higher IA (Fig. 1B–C) are in general agreement with previous reports [7,13,50,[52], [53], [54]]. However, previous studies predominantly emphasized the link of IA with the function and structure of the right anterior insula [4,7,51]. In contrast to these previous findings, there is also evidence associating IA with not only activity of both left and right insula [7,[55], [56], [57]], but also with activity in the posterior and middle insula [52,58]. Furthermore, a recent meta-analysis found predominant association of IA with activity in the left and right posterior insula [59]. One possible explanation for the discrepancy between our findings linking IA with left anterior insula volume and those of previous studies linking IA with right anterior insula volume [7,13] may be related to our sample of only female participants. For instance, in a study investigating differences in the neuroanatomical correlates of IA between female and male participants, the GMV of the left anterior insula was positively associated with IA only among females, whereas the GMV of the left precuneus was positively correlated with IA only among males [60]. In addition, behavioral evidence also suggests the existence of sex differences in heartbeat detection tasks, such as poorer performance by females in heartbeat counting and discrimination tasks [61]. Future studies may contribute to better understanding of how and when distinct insula sub-regions play predominant roles in the emergence of IA, how sex differences in IA come about, as well as how brain, gender, and IA interact and contribute to cognitive processes, or to resilience and vulnerability to the development of psychiatric disorders.

SCN analyses revealed several intra-insula, insula-amygdala, and insula-ACC connections. Granular insula sub-regions together showed the largest number of SCNs with 85 connections (54.84%), followed by the agranular with 43 SCNs (24.74%), and the dysgranular insula with 27 SCNs (17.42%). The large number of identified connections of the granular insula, which serves as the main insular anatomical input region of ascending interoceptive information via the thalamus, suggests that this insula region operates as a hub for the reception and distribution of interoceptive information to other insula regions, the amygdala, and the ACC, an interpretation consistent with neuroanatomical connections and models of insula function [1,6,20,23]. However, the insula not only processes and conveys interoceptive information to other cortical and subcortical systems, but also receives interoceptive information via its mutual connections with the amygdala and ACC [1,20,62,63]. Future studies, possibly with the use of dynamic causal models, may be able to address the directionality of information flow between subcortical and cortical regions involved in interoceptive information processing and IA.

The main finding of the present study is its demonstration of a previously unknown association between IA and intra-insula SCNs. Granular insula sub-regions formed the largest number of SCNs associated with IA (26 SCNs, representing 52% of all its identified connections), whereas only 14 SCNs (28%) of the ventral agranular insula and 10 SCNs (20%) of the dysgranular insula were positively associated with IA (Fig. 3). Although these results suggest that IA may emerge via anatomical connections linking posterior, middle, and anterior insula sub-regions, a finding generally consistent with the actual neuroanatomical organization of the insula [6,20,23,25], the large number of SCNs identified for each insula sub-region and associated with IA may have included false positives from a statistical viewpoint, which could lead to misrepresentation of the insula SCNs with roles in IA. Following a test for multiple comparisons, only the SCNs of the right dorsal agranular insula (seed region) with the left posterior hyper-granular insula and left anterior dorsal agranular insula remained significantly positively associated with IA (Fig. 4). The lack of significant association between IA and the insula-amygdala and insula-ACC SCNs suggests a possible dependence of strengthened neuroanatomical connections linking the posterior and anterior insula for IA. To date, only functional neuroimaging studies have investigated neural networks associated with IA and found either anterior or posterior insula connectivities associated with IA [55,[64], [65], [66]]. The association of IA only with intra-insula posterior and anterior neuroanatomical covariance connections found in the present study contrasts with previous observations linking IA with functional connections of the insula to other cortical and subcortical regions, such as the dorsolateral prefrontal cortex, ACC, supramarginal gyrus and amygdala [52,55,[64], [65], [66]], or with functional networks that did not include the insula as a structure linked with IA [67,68]. These diverse and sometimes contradictory findings indicate that it remains unclear when insula networks are engaged in IA and whether context-dependent recruitment of insula networks may contribute to strengthening and plasticity of its neuroanatomical connections with other structures of the BIN or higher-order regions, such as the prefrontal cortex.

The present demonstration that individuals with stronger anatomical connectivity between posterior and anterior insula exhibit higher IA of heartbeat ascending signals is in general agreement with an elegant hypothesis suggesting that the posterior insula senses visceral signals, e.g., via its thalamic connections, and communicates such changes to the anterior insula, which then integrates these signals with information incoming from other parts of the brain to generate IA [1,4]. However, it has been reported that an interaction between the posterior and anterior insula is not always involved in IA. For instance, decreased functional connectivity between the posterior and anterior insula has been observed among high heartbeat perceivers performing a heartbeat counting task [64]. Furthermore, IA seems to be intact even in patients with insular lesions [[69], [70], [71]]. While the present and previous studies [4,6,13] suggest the importance for IA of interoceptive information flow from visceral-based representations in the posterior insula to higher-order interoceptive representations in the anterior insula, others suggest that IA also depends on other neural pathways, or that lower connectivity and consequent inhibition of interoceptive information flow from the posterior to anterior insula may support IA by reducing interoceptive noise [[69], [70], [71]].

Finally, since lesion studies suggest reduced importance of the insula for IA, could an intact insula still contribute to IA? If so, when and what functions are implemented in insula local structures and networks? We have suggested in our IMAC model that while the brainstem and subcortical nuclei store first-order reflexive innate interoceptive representations and use them to predict desired visceral and physiological states, an intact insula represents second-order cortical interoceptive models that are used not only to predict, but also to learn and map desired visceral and physiological states to context- and experience-dependent behaviors. The IMAC model suggests that insula interoceptive predictions are computed independently by its modular structures (granular, dysgranular and agranular) and supported by their parallel connections with the prefrontal cortex, which stores third-order interoceptive representations. Guided by the active inference framework [30], we suggested that detection of ascending interoceptive information containing interoceptive prediction errors, e.g., abnormalities in visceral and physiological processes, is first processed by a network linking the input region of the insula, the granular insula, with the supplementary motor area (SMA), which uses habit-like interoceptive predictions, e.g., gut feelings, to quickly fix ascending interoceptive prediction errors [6]. On the other hand, if granular insula-SMA network interoceptive predictions fail to fix visceral and physiological disturbances, its interoceptive prediction errors are then sent forward to the anterior insula (dysgranular and agranular), which then implement more flexible active interoceptive inference-based processes, via its connections with the dorsolateral and anterior-medial prefrontal cortices, to identify and introspect on causes of interoceptive prediction errors, giving rising to conscious feelings of body signals. Insula-PFC networks can then generate behaviors and interoceptive predictions that seek to restore homeostasis to visceral and physiological processes. In contrast to a view of passive information flow from the posterior to the anterior insula, the IMAC model offers a more dynamic and integrative framework and suggests the existence of distinct degrees of IA within the insula. While support for the mechanistic microscopic operation of the IMAC model is still lacking, here, we focused our analyses on macroscopic organization of the insula intra-connections and its connections with the amygdala and ACC. Our SCN results support our hypothesis that intra-insula anatomical connections contribute to IA. Future studies need to investigate in detail the mechanistic process of communication between the posterior and anterior insula, possibly with the use of dynamic or granger causal models, as well as how other insula-cortical and insula-subcortical networks contribute to IA.

A few limitations should be acknowledged regarding our sample age, findings and methods. First, our sample was composed of participants of high age, ranging from 40 to 62 years old. We found no relationship between IA and age, a finding that may result from the high age of our participants and contrasts with those of two previous studies reporting an association between reduced interoceptive accuracy and high age [48,49]. In those studies, the age of participants ranged from 22 to 63 years [48] and from 20 to 90 years [49]. Furthermore, due to the absence of younger participants, we could not investigate whether older participants display lower performance, relative to younger participants, in objective measures of IA. Second, although our analyses demonstrated a relationship between IA and state anxiety, we found no relationship between anxiety and insula volume or insula SCN, a finding that contrasted with previous reports linking insula volume, anxiety and depressive symptoms [7,72]. It remains to be investigated whether the lack of association between anxiety and insula SCNs is influenced by gender and age of participants. Third, the SCN method used in this study is an indirect measure of anatomical connectivity based on the correlation between the GMVs of two brain regions. Despite this caveat, SCNs have been shown to be consistent with neuroanatomical networks estimated with diffusion tensor imaging and time-series of functional connectivity neuroimaging data [[43], [44], [45]]. Finally, our focus here was to investigate whether IA is associated with the volume and SCNs of a cortical brain region, the insula. However, it remains an open debate and future studies are needed to investigate whether IA emerges from brainstem and subcortical nuclei, the cerebral cortex, or in neural networks linking them [4,12,73,74].

## 5 Conclusions

The present study tested a hypothesis that brain interoception networks, especially networks centered on the insula, e.g., intra-insula, insula-amygdala, and insula-ACC networks, support interoceptive awareness. Our SCN analyses revealed a previously unknown association of strengthened intra-insula neuroanatomical covariance networks with higher IA. The intra-insula SCNs linked with higher IA involved covariance connections of the right anterior insula (dorsal agranular insula) with the left posterior (hyper-granular insula) and left anterior insula (dorsal agranular insula), suggesting that individuals with stronger anatomical connectivity between the posterior and anterior insula displayed higher IA. Future studies are needed to determine the exact functions of the left and right insula in the emergence of IA, how the insula and its interactions with other regions contribute to conscious and subconscious aspects of interoceptive information processing and how disturbances in insular functions and connections contribute to psychiatric disorders.

## Author contribution statement

Alan S. R. Fermin: Conceived and designed the experiments; Analyzed and interpreted the data; Contributed reagents, materials, analysis tools or data; Wrote the paper, Takafumi Sasaoka, Toru Maekawa, Hui-Ling Chan, Maro G. Machizawa, Go Okada, Yasumasa Okamoto, Shigeto Yamawaki: Analyzed and interpreted the data; Contributed reagents, materials, analysis tools or data; Wrote the paper.

## Data availability statement

Data associated with this study has been deposited at www.openneuro.org/datasets/ds003763/versions/1.0.5.

## Ethics approval and consent to participate

As described in the original publication, the acquisition of data reported here was approved by the local ethics committee of the Research Center of Neurology (Moscow, Russia) and all participants gave written, informed consent. The current study had no access to data that could reveal personal information or identities of the participants.

## Funding

The writing of this manuscript was supported by grants from JST Moonshot-9 JPMJMS2296 to Shigeto Yamawaki and from the 10.13039/501100001691Japan Society for the Promotion of Science (20K07723) to Alan Fermin.

## Declaration of competing interest

The authors declare that they have no known competing financial interests or personal relationships that could have appeared to influence the work reported in this paper.
